# Effectiveness, cost-effectiveness and cost-benefit of a single annual professional intervention for the prevention of childhood dental caries in a remote rural Indigenous community

**DOI:** 10.1186/s12903-015-0076-9

**Published:** 2015-08-29

**Authors:** Ratilal Lalloo, Jeroen Kroon, Ohnmar Tut, Sanjeewa Kularatna, Lisa M. Jamieson, Valda Wallace, Robyn Boase, Surani Fernando, Yvonne Cadet-James, Paul A. Scuffham, Newell W. Johnson

**Affiliations:** Population and Social Health Research Program, Menzies Health Institute Queensland, Griffith University, Gold Coast, Australia; School of Dentistry and Oral Health, Griffith University, Gold Coast, Australia; Australian Research Centre for Population Oral Health, School of Dentistry, The University of Adelaide, Adelaide, Australia; School of Medicine, Griffith University, Logan, Australia; School of Indigenous Australian Studies, James Cook University, Townsville, Australia; School of Dentistry, James Cook University, Cairns, Australia

## Abstract

**Background:**

The aim of the study is to reduce the high prevalence of tooth decay in children in a remote, rural Indigenous community in Australia, by application of a single annual dental preventive intervention. The study seeks to (1) assess the effectiveness of an annual oral health preventive intervention in slowing the incidence of dental caries in children in this community, (2) identify the mediating role of known risk factors for dental caries and (3) assess the cost-effectiveness and cost-benefit of the intervention.

**Methods/design:**

The intervention is novel in that most dental preventive interventions require regular re-application, which is not possible in resource constrained communities. While tooth decay is preventable, self-care and healthy habits are lacking in these communities, placing more emphasis on health services to deliver an effective dental preventive intervention. Importantly, the study will assess cost-benefit and cost-effectiveness for broader implementation across similar communities in Australia and internationally.

**Discussion:**

There is an urgent need to reduce the burden of dental decay in these communities, by implementing effective, cost-effective, feasible and sustainable dental prevention programs. Expected outcomes of this study include improved oral and general health of children within the community; an understanding of the costs associated with the intervention provided, and its comparison with the costs of allowing new lesions to develop, with associated treatment costs. Findings should be generalisable to similar communities around the world.

The research is registered with the Australian New Zealand Clinical Trials Registry (ANZCTR), registration number ACTRN12615000693527; date of registration: 3rd July 2015.

## Background

Globally, of the 50 most prevalent chronic diseases, four are related to oral health: (1) dental caries of permanent teeth, (2) chronic periodontitis, (3) dental caries of deciduous teeth and (4) edentulousness (total tooth loss) [1, 2]. Tooth loss is predominantly a consequence of dental caries in the permanent dentition and adult chronic periodontitis, and potentially results in substantial social and health consequences. Dental caries in the deciduous dentition is a significant predictor of dental caries in the permanent dentition [3]. With few exceptions, both dental caries and periodontitis are preventable conditions with adequate self-care and healthy lifestyle habits, with additional health promotion and preventive interventions at the health services level [4, 5]. However we continue to struggle to significantly reduce, and preferably eradicate, the burden of preventable oral health conditions. Appropriate self-care is largely dependent on the social and health capital of the community [6]. In disadvantaged, marginalised, rural and Indigenous communities, this is often absent. These communities also have the additional burden of limited health promotion and preventive services, carry a significant health burden and suffer a seriously reduced oral health-related, and overall quality of life [7, 8].

Most dental health promotion and preventive services require professionally trained personnel, needing equipment and regular availability of the service for the re-application of interventions. The current evidence is that a number of dental preventive interventions require re-application 2 to 4 times a year [9, 10]; and in disadvantaged and especially remote communities, this is usually not possible, and the burden of the common oral diseases is never dealt with: more importantly, never prevented. To our knowledge there is currently no evidence on the effectiveness, cost-effectiveness and cost-benefit of a less frequent and therefore sustainable dental preventive intervention strategy.

The proposed study will implement a novel dental caries preventive intervention in children to firstly reduce the pathologic bacterial load using an oral anti-septic, secondly seal the part of the tooth most prone to decay with a fissure sealant, and thirdly to strengthen the tooth structure with a fluoride varnish, all during a single annual visit, in a rural remote Indigenous community in Far North Queensland, Australia. While there are data on the effectiveness of the three specific interventions, we have developed a novel approach to assess a less frequent combined application of these interventions. Importantly, we will assess its effectiveness and its cost effectiveness to determine the value for money of the service.

The team believes that the current suggested frequency 2–4 times per year for these preventive interventions is not possible, or sustainable, in poorly-resourced, remote communities and is proposing an alternative strategy requiring fewer resources to address the burden of dental decay in children.

### Burden of dental caries in Indigenous communities in Australia

In Australia, dental conditions are especially prevalent in Indigenous communities, and are a significant health burden in rural remote communities [7, 8]. A recent report of the dental caries status of Indigenous children in Australia showed that those located in rural and/or remote areas have a much higher mean number of decayed, missing and filled deciduous teeth (dmft) (~4 in 6-year old children) compared to non-Indigenous children in metropolitan (dmft ~ 1.5) and rural settings (dmft ~ 1.8) as well as Indigenous metropolitan children (dmft ~ 2.6) [11]. The situation is similar in the permanent dentition of older children. The National Survey of Adult Oral Health found that 57 % of Indigenous adults had untreated coronal dental caries, compared with 25 % of non-Indigenous adults [12]. The mean number of decayed teeth amongst Indigenous adults (>15 years of age) was 2.7 compared to 0.8 amongst non-Indigenous adults.

There are high social costs associated with poor dentition, and a diminished quality of life due to pain and discomfort [13–16], and especially because of long waiting lists for treatment [17]. Social costs include lack of sleep, lost time for school, behavioural problems, lack of cooperation and diminished learning. Lost working time for parents accompanying children to dental treatment sessions has been reported to lead to a loss of employment. Studies in this area need to appreciate the full impact of childhood caries on the child, family and society.

An oral health survey in 2004 (pre-water fluoridation) in the Northern Peninsula Area (NPA) of far north QLD, found that dental caries experience of 6- and 12-year-old children was more than twice that of the state average and more than four times greater than the comparable figures for Australian children overall. Soon after this survey the reticulated water supply of the five small rural communities in this area was fluoridated. A follow-up oral health survey in NPA conducted in November 2012 by this team, in which we examined over 70 % of known resident schoolchildren (*n* = 339), suggests that the dental caries status has improved significantly since the 2004 survey [18]. Few teeth had restorations in both surveys. Age-weighted overall caries prevalence and severity declined from 2004 to 2012 by 37.3 %. The effect was most marked in younger children, dmft decreasing by approximately 50 % for ages 4 to 9 years; at age six, mean decayed score decreased from 5.20 to 3.43. DMFT levels decreased by half in 6 to 9 year old children. However, significant unmet treatment needs exist at all ages. To address this, practical and affordable ways have to be found.

One of the reasons for the improved oral health status could be due to fluoridation of the local water supplies. Whilst the economic viability of water fluoridation for a small community such as this might be questioned, we posit the costs are outweighed by the significant caries reduction in both the deciduous and permanent dentitions as found in our study [18]. Moreover, the fluoridation plant has functioned erratically since being implemented and has been out of operation since April 2011 following a lightning strike. The likelihood that the water will again be fluoridated is uncertain due to budget constraints and because the 2012–2015 Queensland State Government legislated to give local governments across the State the power to decide to fluoridate or not. Dental caries rates may again increase in the absence of water fluoridation. It will be crucial to investigate alternative models to ensure improvements in dental caries incidence in this resource-constrained community. The envisaged dental prevention model will essentially reduce the microbial load with the topical disinfectant, povidone iodine [10]; inhibit biofilm adherence to susceptible sites by application of fissure sealants [19] and reduce the susceptibility of the tooth to demineralisation by acids generated in the microbial biofilm by the application of a fluoride varnish [9, 20]. Measures of local fluoride concentration [20] and of oral mutans streptococci counts return to baseline after a few months [21], so that most reported studies have used regular re-applications (2–4 times per annum), which would be difficult to implement in remote settings lacking appropriately trained personnel and resources. Fissure sealants, on the other hand, have excellent longevity [19].

To date, the reported economic burden of childhood caries is likely to be underestimated, as previous reports did not capture the full scope of costs and missed the potential cost-savings of prevention programs. There are few studies involving child populations that evaluate the cost-effectiveness of prevention programs for childhood caries [22]. Savage et al. [23] concluded that pre-school aged children in the USA who had early dental prevention visits would experience lower dental-related costs over 5 years. Similarly, a second USA study by Ramoz-Gomez et al. [24] looked at minimal, intermediate and comprehensive prevention programs and concluded that all three were cost-effective. They concluded that government health systems can save considerable resources by investing in early childhood caries prevention. A study by Lee et al. [25] in Carolina, USA, found early dental visits were highly cost-effective for high-risk children. These studies reiterate the need for translating this evidence into policies for childhood caries prevention. Although the cost-savings of prevention programs are based on predictions, the potential economic benefits are encouraging.

### Review of interventions to reduce the incidence of dental caries in children

#### Pit and fissure sealants

Dental decay most often occurs on the occlusal pits and fissures of permanent molar teeth. A pit and fissure sealant is defined as a material [both glass-ionomer and resin-based materials are widely used] that is introduced into the occlusal pits and fissures of caries susceptible teeth [19]. Its application to newly-erupted posterior teeth is the best method to prevent pit and fissure caries, and/or to prevent the continued development of incipient caries into frank caries when the incipient lesion is sealed over. Sealing the occlusal surfaces of permanent molars in children and adolescents reduces the incidence of new carious lesions for up to 48 months when compared to no sealant. Recommendations and reviews of the evidence of preventing dental caries through school-based sealant programs suggest that it remains an important and effective public health approach [26].

#### Fluoride varnish

Fluoride varnishes are a liquid resin or synthetic base that contain a high concentration of fluoride and set quickly on contact with teeth, even in the presence of saliva [9]. Fluoride ions in the material are released when the pH drops in response to acid production in the biofilm on the tooth surface and these become available to promote remineralisation of damaged tooth enamel in early carious lesions (white spots). The fluorhydroxyapatite formed over time during the remineralisation process in an initial caries lesion is more resistant to future demineralisation. Children at moderate or high-risk to dental caries benefit from fluoride varnish programs. Varnish delivers a higher concentration of fluoride than other professionally applied fluoride gels and foams; therefore it is applied in smaller amounts.

The fluoride varnish layer slowly disappears over time and needs repeated application to maintain effectiveness as a primary prevention strategy. While one application of fluoride varnish may provide some benefit, the majority of studies of professionally applied fluoride demonstrate that at least two applications each year, for at least 2 years, are necessary to demonstrate effective reductions in dental caries, making this a difficult strategy in disadvantaged communities who are most in need of this intervention [20].

#### Povidone (PVP)-iodine addition to fluoride

Children with high rates of tooth decay are much more heavily colonized with pathogenic bacteria than children who experience less tooth decay [27]. PVP-iodine interferes with the ability of *mutans streptococci* to bind to tooth surfaces by disrupting the expression and production of glucosyltransferase [28]. A seminal in vitro study of anti-plaque agents demonstrated that 1 % iodine (10 % PVP-iodine is 1 % active iodine) was bactericidal to intact *S. mutans* biofilm [29]. A series of studies have led scientists to conclude PVP-iodine is an appropriate adjunctive antimicrobial for use on teeth to prevent tooth decay [10, 21, 30, 31].

Topical Treatment by PVP-Iodine in conjunction with Fluoride Varnish is simple [32]. The iodine comes in a single application swab. The total treatment time is 3 to 4 min and costs less than 20 cents. Clinically the teeth are brushed to remove debris and disrupt the biofilm; then dried with gauze, and painted with 0.2 ml PVP-Iodine. After the iodine application, the teeth are dried again and coated with fluoride varnish at the same visit.

As a result of our recently conducted oral health survey in the NPA, the research team has established networks and support from the local community, health workers and schools, as well as from Queensland Health (QH), the State agency responsible for all public health services. The importance of sustainable dental prevention interventions is evident, especially if fluoridation of the water supplies is not to continue. This study will assess the effectiveness, cost-effectiveness and benefits of a novel single annual dental caries preventive intervention in a remote rural Indigenous community in north Queensland.

### Aims and hypotheses

The study seeks to (1) assess the effectiveness of an annual oral health preventive intervention in slowing the incidence of dental caries in children in a remote, rural Indigenous setting, (2) identify the mediating role of known risk factors for dental caries and (3) assess the cost-effectiveness and cost-benefit of the intervention.

We hypothesise that 1 and 2 years after the intervention (1) the actual incidence of dental caries in children will be significantly lower than the expected incidence, based on modelling from the two oral health surveys conducted over the past 11 years in the same community, the current survey and (2) the intervention will be cost-effective and cost beneficial, and therefore feasible and sustainable for broader implementation across similar communities in Australia and internationally.

## Methods

### Study design

This is a longitudinal preventive intervention study. All school children in the NPA will be invited to participate. As it is unethical to withhold any proven intervention from any child, no control group will be created. Children who do not consent to participate may be natural controls if they consent to a dental examination at the end of the study. The actual caries increment in the children who participate will be compared to the expected caries increment modelled on oral health surveys carried out in this community in 2004 (pre-water fluoridation); in 2012 (by the Griffith University team post-partial water fluoridation) and 2015 (baseline survey for this study).

All consenting children will undergo a detailed head, neck and dental clinical examination and complete a questionnaire on their basic demography (gender and age), residential history (exposure to fluoridated drinking water), own general and oral health perceptions; oral health behaviours, attitudes and knowledge; dental visits; diet and oral health-related quality of life.

All active disease will be treated prior to implementing the dental caries preventive intervention. In years 2 and 3 of the study, all participating children will be invited to return for a dental examination, treatment of new incident disease and repeat of the prevention regime.

### Study setting

The study will be conducted in a number of small towns in the remote Northern Peninsula Area (NPA) of Far North Queensland. In the 2011 Census the population of the NPA was estimated at 1046 and is comprised of 52.8 % females and 47.2 % males. The median/average age of the NPA population is 22 years, 15 years below the Australian average. 98.7 % of people living in these communities were born in Australia.

English is spoken as first language by 22.7 % of the population, Yumplatok (Torres Strait Creole) by 64.9 %, Kalaw Kawaw Ya/Kalaw Lagaw Ya by 1.8 and 0.3 %. Sixty eight percent of residents are employed full time, and 21 % are working on a part time basis. The NPA has an unemployment rate of 7 %. The median individual income is AUD554.00 per week and the median household income is AUD1, 179.00 per week (AUD = Australian Dollar).

### Study participants

All children (approximately 600–650) attending the two primary and one secondary school campuses will be invited to participate in the intervention study. These children will be 4–17 years of age, and almost all are Indigenous. As all children in the community will be invited to participate a sample power calculation was not performed.

### Intervention

The proposed “Big Bang” preventive intervention is to firstly reduce the pathogenic bacterial load, then seal grooves on posterior (molar) teeth, and thirdly to strengthen the tooth structure.

Oral health promotion will essentially educate children regarding appropriate health behaviours to maintain oral health, with emphasis on the importance of toothbrushing with fluoridated toothpaste, healthy eating habits, emphasising the role of sugar in the tooth decay process, and the importance of reducing both the quantity and frequency of sugary products in the diet.

Prior to the annual intervention the research team will undertake clinical examination of all consenting school-age children to assess dental caries experience. A team comprising of a dentist and/or oral health therapist will treat all existing tooth decay and other oral health problems. Each child will receive the preventive intervention when other treatments are completed.

Prior to the treatment of existing disease, we will investigate saliva of the participants as a component of caries risk assessment. Saliva will be collected using commercially available test kits for measurement of flow rate, pH, buffering capacity and then cultured for bacterial assessment [33]. The number of teeth, fillings and other retentive sites in mouth influence the bacterial load and a high count of bacteria in dental plaque correlates with salivary bacterial counts, making it possible to assess saliva for cariogenic microbes [34, 35]. Such kits use selective media for mutans streptococci and for lactobacilli.

### Primary outcome variable

The International Caries Detection and Assessment system (ICDAS-II) for clinical caries diagnosis will be used to record caries experience and to determine incidence [36]. This will be measured annually across the 3 years of the study, at baseline and after years 2 and 3 of the project.

ICDAS-II consolidates features of several caries classification systems into one universal system using a six-point ordinal scale ranging from non-cavitated to extensive cavitated lesions to describe any signs of past or present caries activity. Unlike World Health Organisation (WHO) caries detection criteria, ICDAS-II allows the detection of initial/non-cavitated carious lesions, and is thus considerably more sensitive, whilst allowing the extraction of data comparable to previous surveys which used WHO criteria [37].

### Secondary outcome variables

General Child Quality of Life, the social impact of oral disorders and Oral Health-Related Quality of Life (OHRQoL) will be measured at baseline and at years 2 and 3. Existing validated and reliable instruments will be used (CHU-9D [38], OHIP-14 [39] and Child-OIPD [40]), appropriately modified for our participants.

The retention of the fissure sealants at the follow-up periods will be assessed, and recorded. Saliva of the participants will be collected again in years 2 and 3. Findings will be compared to baseline to assess the impact of the less frequently applied anti-bacterial component of the intervention.

Resources use and costs of providing the intervention will be recorded throughout the intervention period. Resource use and costs to participants to receive the intervention (e.g. time off work to bring the child to the clinic) will be recorded as well as any emergency treatment required between annual visits by the team. Emergency treatment includes travel to the nearest dental facility, the Royal Flying Doctors Service call-outs, local GP visits for antibiotics and analgesia, and so forth.

### Covariates

The relationship between the primary and secondary outcomes and the preventive intervention will be adjusted for known risk factors for dental caries - oral hygiene behaviours and diet (sugar consumption).

### Analysis

All baseline socio-demographic characteristics will be described for the selected sample using counts and frequencies. Baseline and follow-up caries experience and questionnaire related information will be reported. Dental caries increment (incidence) will be the main outcome measure used to determine the effectiveness of the preventive intervention. The expected caries increment will be modelled from the three oral health surveys conducted in this community (2004; 2012 and 2015) and compared with the actual caries increment from 2015–2016; 2016–2017 and 2015–2017. The mean caries increment will be compared between the expected (modelled) and actual findings, and adjusted for known risk factors for dental caries. The hypothesis will be that caries increment observed in the period of 2015 to 2017 is smaller than the modelled caries increments. Two independent samples *t*-test will be used for the analysis with significance being determined if *p* < 0.05.

Children who receive only a part of the intervention will be separately assessed: for example we will have children who fully participate, those with baseline and only a year 1 follow-up, those with baseline and only a year 2 follow-up. This will ‘naturally’ further inform us on the most appropriate frequency of this preventive strategy. Both a group and matched analysis will be conducted to account for children who receive only part of the intervention.

#### Development of Markov model

A health state transition Markov model will be developed using Tree Age pro software (TreeAge Software Inc., Williamstown, Massachusetts, USA) to analyse the cost effectiveness of the intervention. The model will be populated with the caries experience of the children in NPA using the intervention caries data and compared with modelled data from baseline in a non-intervention scenario. The model cohort will start at the age of 6 years where mixed dentition is emerging. The model will track these children up to 17 years using the data from the study for each year. Health states for the model will include “healthy” and “caries” health states. It is anticipated to have health states for conditions such as pulpal abscess as well. The model will be made sensitive for waiting periods, available treatment facilities, availability and costs of resident or fly-in/fly-out professional staff and common practices of the local dental clinics.

#### Costs calculations

The costs of providing the preventive intervention, the costs of all treatment for carious lesions, and the out-of-pocket costs in relation to caries experience will be assessed. These costs will be assigned to each child taking into account the number of surfaces treated. Cost intervention will include sealants, an oral anti-septic application, application of a fluoride varnish and including cost for human resources and logistics. The costs of treating incremental caries will be estimated using government costs for treatments. Total out-of-pocket costs for parents of children with caries will be calculated based on the quantities of resource use provided in the surveys. Mean, median and interquartile range costs will be presented for each major treatment category in caries. All costs will be presented in 2015 AUD.

#### Estimation of utility weights

The utility values for dental health states will be estimated from the CHU-9D data. Using the CHU-9D (Child Health Utility) multi attribute utility instrument, quality of life scores (utility scores) for each caries severity level experienced by the children will be determined. A scoring algorithm that has been validated in the UK for CHU-9D for children will be applied [38]. Utility scores will be presented for different age groups and gender. These values will contribute to estimate Quality Adjusted Life Years (QALYs). The CHU-9D will be validated in a similar Indigenous population prior to the application in the study population.

#### Transition probabilities

Caries increment prior to intervention and post intervention will be used to calculate transition probabilities respectively for the two scenarios of non-intervention and intervention examined by the Markov model. The caries increment rates for intervention will be directly observed. These rates for the non-intervention will be estimated from the modelled data.

#### Cost utility analysis

The cost utility of the “Big Bang” prevention strategy will be estimated using the Markov model. This analysis will adhere to the best modelling practices as given by International Society for Pharmacoeconomics and Outcomes Research (ISPOR) guidelines [41]. All costs will be presented in 2015 AUD. Costs and outcomes will be discounted at 5 % per year. The model will present the societal perspective. Incremental cost effectiveness ratio (ICER) will be generated by calculating incremental costs for caries treatment divided by the outcome (number of carious lesions prevented and QALYs gained separately). The intervention group will be compared with the modelled values for a non-intervention scenario. To address the uncertainty in the costs and effectiveness estimates, univariate sensitivity analyses will be used. For all probabilities, the 95 % confidence intervals will be used, and for costs high and low values will be estimated. A probabilistic sensitivity analysis will also be performed by re-sampling 1000 times at random from the probability distributions for each parameter. This procedure is similar to multivariate sensitivity analysis and will address the uncertainty of all estimates simultaneously. Gamma distributions will be used for cost estimates and beta distributions will be used for probabilities.

### Ethics

Prior to seeking ethics approval for the project, support was obtained from the QH Chief Dental Officer (CDO), community Elders, Cape York Health Council (Apunipima), the Torres and Cape Health and Hospital Service (TCHHS) management of QH, the Northern Peninsula Area Regional Council (NPARC) and the principal of the Northern Peninsula Area State College (NPASC).

Ethics approval was primarily sought from the Griffith University Human Research Ethics Committee (GUHREC) though a National Ethics Application Form (NEAF) submission, taking into account the “National Statement, Values and Ethics: Guidelines for Ethical Conduct in Aboriginal and Torres Strait Islander Health Research” and the “Australian Institute of Aboriginal and Torres Strait Islander Studies (AIATSIS) Guidelines for Ethical Research in Indigenous Studies” [42, 43]. Following approval from the GUHREC, a further ethics approval submission was made to the Far North Queensland Human Research Ethics Committee (FNQHREC). Both committees have approved the study protocol.

An information sheet as per the template of the GUHREC will accompany the informed consent form to the parents/guardians of potential participants for approval prior to examination, treatment and any preventive intervention being performed.

## Discussion

Tooth decay in rural Indigenous children is unacceptably high, and their general and oral health-related quality of life is significantly compromised. There is an urgent need to reduce the burden of dental decay in these communities, by implementing effective, cost-effective, feasible and sustainable dental prevention programs. Expected outcomes of this study include improved oral and general health of children within the community; an understanding of the costs associated with the intervention provided, and its comparison with the costs of allowing new lesions to develop, with associated treatment costs. The work will benefit Indigenous children and reduce disparities. If found to be effective and cost-effective in reducing dental caries, this initiative could be implemented in similar communities elsewhere.

The study will provide longitudinal data on dental caries prevalence and incidence and OHRQoL for remote Indigenous children, a group often neglected or under-represented in national oral health surveys. The study will further provide information on the oral hygiene practices and diet (sugar) of these children. Data on the impact of a less frequent antibacterial intervention on the presence of the main dental caries associated bacteria in the saliva, as well as the retention of fissure sealants, will be reported.

## Abbreviations

AIATSIS: Australian Institute of Aboriginal and Torres Strait Islander Studies
ANZCTR: Australian New Zealand Clinical Trials Registry
AUD: Australian Dollar
CDO: Chief Dental Officer
Child-OIDP: Child-oral impact on daily performance
CHU-9D: Child health utility-9D
dmft: Decayed, missing, filled deciduous teeth
DMFT: Decayed, missing, filled permanent teeth
FNQHREC: Far North Queensland Human Research Ethics Committee
GP: General practitioner
GUHREC: Griffith University Human Research Ethics Committee
ICDAS: International Caries Detection and Assessment system
ICER: Incremental cost effectiveness ratio
ISPOR: International Society for Pharmacoeconomics and Outcomes Research
NEAF: National Ethics Application Form
NHMRC: National Health and Medical Research Council
NPA: Northern Peninsula Area
NPARC: Northern Peninsula Area Regional Council
NPASC: Northern Peninsula Area State College
OHIP-14: Oral health impact profile-14
OHRQoL: Oral health-related quality of life
PVP: Polyvinylpyrrolidone
QALY: Quality Adjusted Life Years
QH: Queensland Health
QLD: Queensland
TCHHS: Torres and cape health and hospital services
WHO: World Health Organisation
